# Laryngectomy Education and Training Resources: An Environmental Scan of Resources

**DOI:** 10.1007/s13187-025-02606-1

**Published:** 2025-06-11

**Authors:** Laura-Jayne Watson, Emer Fahy, David W. Hamilton, Linda Sharp, Vicky Thornton, Joanne M. Patterson

**Affiliations:** 1 University of Liverpool, Liverpool, UK; 2 South Tyneside and Sunderland NHS Foundation Trust, Sunderland, UK; 3 Speech and Language Therapy, The Clatterbridge Cancer Centre, Liverpool, UK; 4 Head and Neck Surgery, Freeman Hospital, Newcastle Upon Tyne, UK; 5 Population Health Sciences Institute, Newcastle University Centre for Cancer, Newcastle University, Newcastle Upon Tyne, UK; 6 Institute of Population Health, University of Liverpool, Liverpool, UK; 7 School of Allied Health Professions & Nursing, Institute of Population Health / Liverpool Head and Neck Centre, University of Liverpool, Liverpool, UK

**Keywords:** Laryngectomy, Education and training, Community care

## Abstract

In many settings, head and neck cancer (HNC) services are centralized in large hospitals, meaning that community input from HNC specialists can be lacking. Community healthcare professionals often require laryngectomy-specific education and training to provide optimal care to people at home. However, laryngectomy-specific education/training is often sporadic and varies between centres, making it difficult to achieve standardisation in healthcare professionals’ laryngectomy knowledge and skills. This study aimed to identify and critically review existing laryngectomy education/training resources for healthcare professionals, which include elements applicable to community care. We used environmental scanning to systematically and comprehensively identify and examine all available education/training resources which had an element of education and/or training applicable to community laryngectomy care. Eligible resources were reviewed for training-specific details, laryngectomy-specific details, evaluation method, accessibility, and patient and public involvement (PPI). A total of 6655 resources were screened; 49 were eligible for data extraction. There was wide variability in the level of laryngectomy-specific content in the resources. PPI in development was lacking, and data detailing the impact of resources on healthcare professionals’ knowledge/skills and patient outcomes was sparse. This is the first study to systematically identify and appraise laryngectomy education/training resources which have applicability to community care. There is not one consistent approach to education/training development method, content, delivery, or evaluation, likely rendering these ineffective and lacking impact. Community-specific laryngectomy education and training, with clear implementation and evaluation, is required.

## Introduction

Treatment for advanced laryngeal cancer often requires total surgical removal of the voice box, known as a total laryngectomy [1]. Those who undergo laryngectomy have a high need of support as they experience profound changes to everyday functions [2] including permanent loss of their natural voice and life-long changes to swallowing, breathing through a permanent stoma at the front of the neck, and appearance [3].

Post-laryngectomy, people need to learn complex new skills such as looking after their new airway safely and caring for surgical voice restoration (a silicone valve inserted into the wall of muscle between the trachea and oesophagus to allow pseudo-voice production) in the early post-operative period. This is already a highly distressing time [4], but it is essential to enable ‘safe’ discharge home. It is common that people experience significant challenges in acquiring these skills, which may be explained by the link to advanced laryngeal cancer being strongly socio-economically patterned [5], which is often associated with poorer health literacy [2]. As such, it is commonplace that people after laryngectomy often need help from their family/friends for supporting practical skills, emotional adjustment, navigating health services, and liaising with healthcare professionals (HCPs) [6].

Community healthcare is vital in supporting individuals and their families once they are at home to aid ongoing laryngectomy skill confidence, safety, and psycho-social adjustment [6]; however, there is often a stark lack of community healthcare intervention and support for people post-laryngectomy. This intervention is particularly important as most people after laryngectomy are over 70 years old, and a third either live alone or in a care facility [7]. Consequently, this often leads to a high hospital re-admission rate (10%) [7]; re-admission risk is higher for those requiring long-term airway management and psycho-social care [8, 9].

Head and neck cancer (HNC) services in healthcare settings are most often centralized in large hospitals, meaning that direct community input from HNC specialist HCPs is not readily available, putting significant pressure on community staff who often have limited time, busy caseloads and lack specialist laryngectomy skills, knowledge, or training [10]. Additionally, the move towards enhanced recovery after surgery (ERAS) programs to reduce hospital length-of-stay [11–15] could add to the burden on community healthcare teams. Patients feel that community-based HCPs are important throughout their cancer pathway [16–18]. However, data suggests that as few as 5% of front-line emergency staff can describe basic laryngectomy anatomy [19], with 65% of UK primary and secondary care nurses reporting inadequate stoma care training [20]. Complication rates with laryngectomy stomas in the community in Australia are reported as being as high as 60% [21], although the need for better education is recognised [16, 21]. Moreover, only 43% of laryngectomy patients consider community HCPs to be adequately trained to manage their needs [22]. To add to this problem, laryngectomy-specific education and training are often sporadic, inconsistent, and variable, making it difficult to achieve standardisation in HCP laryngectomy knowledge and skills. This stark lack of training combined with low levels of confidence in community HCPs from patients themselves [23] poses significant safety concerns for people in the community regularly accessing healthcare from non-HNC specialist HCPs.

It is imperative that community HCPs can access community-specific laryngectomy education and training to better meet the needs of people after laryngectomy [23, 24]. This study identifies and critically reviews the existing laryngectomy education and training resources for HCPs, with a focus on community services. This will aim to highlight the gaps in laryngectomy education and training designed for community HCPs to improve the safety and care of laryngectomy patients at home.

## Methods

An environmental scanning method was used to identify and review the existing laryngectomy education and training resources designed for HCPs. Environmental scanning is a recognised technique in health services research often used to examine the landscape by drawing on information gathered from internal and external environments [25–29] to allow the examination of a broad range of issues. Information is gathered systematically from a variety of sources [30] to comprehensively identify and map resources and gaps [29, 31].

Choo’s framework for effective environmental scanning and analysis [32] was used to inform the selection of the mode of scanning. We used the active searching mode, as this allowed systematic searching and focussed information gathering on the topic at hand. Specifically, we applied a mixed approach to comprehensively identify resources, including electronic searches of available education and training resources and grey literature, social media scanning, targeted websites, and consultation with key informants.

The environmental scan followed an iterative design, with preliminary findings discussed as part of the consultation with key informants involved in the process. Following this process, the certification status of the resources was added to the data extraction, resources which did not reference any evidence were classified as ‘no evidence base’, and patient and public involvement (PPI) involvement in the design and/or delivery of the resources was specified. Key informants included the research team, an expert HCP advisory group, and a PPI group.

### Eligibility Criteria

We sought to identify all education and training resources which had an element of education and/or training applicable to HCPs providing laryngectomy care in community settings anywhere in the world and were available in English. This broad criterion, with no date or geographical limits, allowed for a wide range of resources to be screened for potential inclusion in the scan. Education and training resources, focussing on tracheostomy only, or unavailable in English were excluded.

### Expert Consultation

We consulted with expert clinicians experienced in developing laryngectomy education and training identified through our research networks and/or from Twitter/ ‘X’ searches. These clinicians were contacted via personal email invitation from the lead author and a follow-up call using Microsoft Teams with those who responded. This consultation supported the decision-making process around which topics to include in data extraction and subsequent analysis, for example, accessibility of education/training programme, and directed the researcher to any other available education and/or training resources for eligibility screening.

### Search Strategy

The scan was conducted by the lead author (LJW) between September 2023 and January 2024. This included an electronic search of available education and training resources and grey literature, conducted with support from information specialists at the University of Liverpool, using the keywords ‘laryngectomy’, ‘community healthcare’, ‘education’, and ‘training’. Structured searches were conducted via Google and literature databases including OpenGrey, Web of Science, Scopus, PubMed, PsychInfo, AMED, NHS Knowledge & Library Hub, TRIP medical database, and Social Science Research Network. Google searches were limited to the first 100 results per search [33]. Targeted websites that we knew had education and training: ‘The National Association of Laryngectomy Clubs’ (NALC) [34] and ‘The National Tracheostomy Safety Project’ (NTSP) [35] were searched. We also conducted searches of the social media network, Twitter/‘X’, following a data collection approach using pre-determined keywords and hashtags [36]. Keywords and hashtags used were ‘community laryngectomy education’, ‘community laryngectomy training’, ‘community laryngectomy healthcare’, ‘#laryngectomy’, #community laryngectomy’, ‘#laryngectomy training’, ‘#laryngectomy education’.

All literature/resources retrieved via the searches were screened via abstracts, executive summaries, or table of contents prior to full-text screening for resources that were judged potentially eligible [37]. References/links identified from the searches were hand-searched to screen for any other resources.

### Data Extraction

Information about the identified resources was comprehensively reviewed using a data extraction form. This allowed the extraction of relevant data about the development, content, evaluation, and accessibility of education and training. The mode of training was characterized using the following pre-agreed categories: case studies, coaching, eLearning, instructor-led training, interactive training, on-the-job training, video-based training, and information-based training, most often based on clinical expertise. All the resources were reviewed for their laryngectomy-specific details. Some education and/or training resources required a paid premium, with only an overview of the information available freely. Therefore, information was extracted based on the freely available information for some of the resources included.

The data extraction form was devised by the lead author and informed by expert consultation, PPI involvement, Perryman’s training intervention taxonomy [38, 39], and the TIDier framework [40]. The level of training evaluation as per the Kirkpatrick model [41] which provides a structured approach to evaluation using four training levels from reaction to learning to behaviour to results was also included.

Data extraction was conducted by two independent reviewers (LJW and EF), with discrepancies discussed to achieve consensus. Where information was ‘missing’, for example, who the training was developed by, a relevant individual, such as the lead author on paper publications, was contacted. The final content of the consensus data was reviewed with the research team, the HCP advisory group, and the PPI group.

### Data Synthesis

The final content of the consensus data was then entered into a matrix to enable synthesis and comparisons. The content was grouped into four categories in the matrix: (1) training origin and audience, (2) training development and resources, (3) laryngectomy-specific details, (4) evaluation, accessibility, and PPI. Synthesis of the extracted data informed by a narrative synthesis approach [42] and content analysis [43] was used to draw out commonalities in the training as well as identify gaps.

## Results

**Fig. 1:**
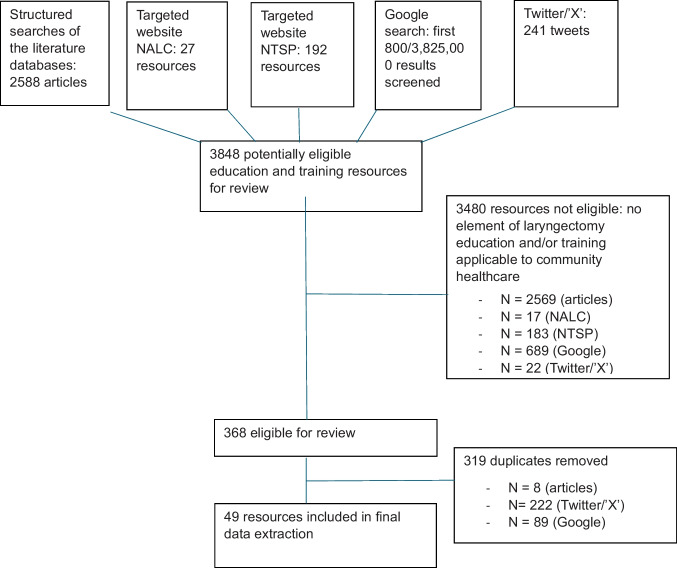
Flow scheme of the environmental scan

In total, the search yielded 6655 potential resources. Of these, 3480 were ineligible and 319 duplicates were removed, leaving 49 eligible for data extraction from the following sources: 10 from the UK National Association of Laryngectomy Clubs (NALC) [34] [44–53], 8 from the National Tracheostomy Safety Project (NTSP) [35] [54–61], 20 Google search results [62–81] (2 of which were also identified on Twitter/X), and 11 journal articles [82–92]. No additional eligible articles were found via hand-searching. Figure 1 displays the flow scheme of the environmental scan.

Forty-four education and/or training resources were developed in either the UK (*n* = 26) [44–61, 63, 64, 66, 73, 77, 80, 90, 91], USA (*n* = 17) [62, 65, 67–69, 72, 74–76, 78, 82, 83, 85–87, 89, 92], or joint from the UK and USA (*n* = 1) [70]. The remaining five resources were from Australia (*n* = 2) [71, 80] or Canada (*n* = 3) [79, 84, 88]. A similar pattern was seen for the geographical target: resources were intended for HCPs in the location in which they were developed; the exceptions were two resources developed in the UK/USA which were intended for “global” use [63, 74] and one UK-developed resource which did not state a geographical target [77]. The target audience for 26 resources were HCPs in general [44, 53, 54, 59, 62, 64, 67–70, 73–75, 77, 80–82, 87, 89, 90, 92], including trainee medical and nursing staff, doctors, and speech and language therapists. Eleven resources stated that they were aiming towards a specified HCP group only: first responders (*n* = 5) [45, 47–50] and nurses (*n* = 6) [46, 51, 66, 72, 88, 91]. One resource was targeted towards both HCPs and patients [63] and 11 resources did not state their target audience [52, 55–58, 60, 61, 71, 76, 78, 79, 81].

### Training Development and Resources

Over one-third of education and/or training resources were developed by either NALC or NTSP (*n* = 18) [44–61]. The method of development for 43 resources was not stated [44–66, 68–72, 74–81, 84–87, 90–92. Only six resources used literature reviews and clinical expertise [67, 73, 82, 83, 88, 89]. The NTSP resource manual was developed in consultation with key national bodies, stakeholders, and case reports but does not specify the method used for each resource. Twenty-two resources were based on guidelines and peer-reviewed literature [54, 56, 58–60, 62–64, 73, 74, 76–78, 84–90]. Despite this, no resources included in this scan utilised theoretical models when developing their education/training. Seventeen resources specify learning objectives for the user—some with one over-arching objective and others with specific objectives focussed on smaller topics [59, 60, 62–65, 67–73, 75, 78, 82, 89].

The method of training was classified into the following categories: video-based, e-learning, information-based, interactive, and instructor-led. Forty-two resources were in a single category: video-based (*n* = 11) [44–49, 52, 61, 65, 74, 80], information-based (*n* = 10) [50, 51, 53–58, 76, 79], eLearning (*n* = 7) [59, 60, 68, 69, 71, 75, 88], interactive (*n* = 4) [67, 70, 78, 86], and instructor-led (*n* = 10) [64, 66, 72, 81, 85, 87, 89–92]. Of the remaining seven resources, six were categorised into more than one category [62, 63, 73, 77, 82, 83], and one resource’s method of training was undefined [84].

A range of training resources were used to deliver the education/training including lectures, animation, audio, written text, visuals, simulation, clinical vignettes, and virtual reality. Forty-four resources used more than one training resource, with only four resources relying on one training resource [66, 72, 74, 78]. Nineteen resources used two training resources, for example, lecture with video or written information with visuals [45, 47, 50–52, 61, 65, 75–77, 79, 80, 86, 91]; 11 resources used three training resources, for example, videos, animation, and pictures/diagrams [44, 58, 62, 68, 69, 83, 88–90, 92] and 12 resources used four or more training resources including lecture with video, animation and patient demonstration or lectures/theory, virtual reality, simulation, and clinical review [46, 48, 49, 59, 60, 63, 64, 67, 70, 73, 82, 87]. The training resources were unknown/not available for three resources [71, 82, 82].

### Laryngectomy Specific Details

Table 1 summarises these details and information included in the resources.

**Table 1:** Summary of laryngectomy specific detail and content

Package	Anatomy and physiology	Airway	Communication	Swallow	Psycho-social (patient)	Psycho-social (family)	SVR	Equipment	Emergency management (community)	Emergency management (acute)
NALC Speech after laryngectomy [44]	✓ Communication only	X	✓	x	X	x	x	x	x	x
NALC Resuscitation [45]	✓ Airway only	✓	x	x	X	x	x	✓	✓	x
NALC laryngectomy nursing, part 1 and 2 [46]	✓	✓	✓	✓	✓	✓	✓	✓	✓	x
NALC Laryngectomee Emergencies [47]	✓	✓	✓	x	X	x	x	✓	✓	x
NALC Laryngectomy Info for First Responders [48]	✓	✓	✓	✓	X	x	x	✓	✓	x
NALC Rescue Breathing for Neckbreathers [49]	X	✓	X	x	X	x	x	✓	✓	x
NALC Neck breather Emergency Care Leaflet [50]	✓	✓	X	x	X	x	x	✓	✓	x Community information applicable
NALC Nursing Care Leaflet [51]	✓	✓	✓	✓	✓	✓	✓	✓	x	x
NALC – YouTube [52]	✓ Airway only	✓	✓	X	✓	x	x	x	x	x
Laryngectomy Information Sheet (NALC/NTSP) [53]	✓	✓	✓	X	X	x	✓ Leaving in situ in emergency only	✓	x	✓
NTSP Emergency Laryngectomy Management [54]	✓	✓	X	X	X	x	x	✓	✓ Acute information applicable	✓
NTSP Humidification [55]	✓	✓	X	x	x	x	x	✓	x	x
NTSP Laryngectomy Humidification [56]	✓	✓	X	x	x	x	X	✓ Humidification only	x	x
NTSP Speech after Laryngectomy [57]	X	X	✓	x	x	x	✓ Leaving in situ in emergency only	✓	x	x
NTSP Suctioning [58]	X	✓	X	x	x	x	x	✓	x	x
NTSP Module 3 [59]	✓ Airway only	✓	X	x	x	x	x	✓	x	x
NTSP Module 5 [60]	✓	✓	✓	x	✓	x	✓	✓	✓	✓
NTSP Assess the patency of a laryngectomy stoma – YouTube [61]	X	✓	✓	x	x	x	x	✓	✓	✓
Laryngectomy | Tracheostomy Education [62]	✓	✓	✓	✓	x	x	✓ TEP loss only	✓	x	✓
ATOS learning institute [63]	*	*	*	*	*	*	*	*	*	*
Sparks [64]	✓	✓	x	x	x	x	✓	x	x	✓
Laryngectomy Interdisciplinary Education [65]	✓	✓	X	x	x	x	✓ Limited to “what is a TEP?”	✓	x	x
ENT | Nursing Times [66]	✓	✓	✓	x	x	x	✓ Emergency management only	x	x Airway emergency section but unclear as to environment	x Airway emergency section but unclear as to environment
Jeff Searl cancer grant [67]	✓	✓	X	x	x	x	✓	✓	✓	✓
MedBridge (1) [68]	✓	✓	✓	✓	x	x	x	✓	x	x
MedBridge (2) [69]	x	✓	✓	✓	✓	x	✓	x	x	x
GP Courses [70]	✓	✓	X	✓	x	x	x	✓ Limited to tubes	x	x
Care of adult patients with a tracheostomy or laryngectomy [71]	**	**	**	**	**	**	**	**	**	**
UC Davis [72]	**	**	**	**	**	**	**	**	**	**
Reynolds [73]	X	✓	✓	✓	x	x	x	✓	x	✓
Inhealth [74]	✓	✓	✓	✓	✓	x	✓	✓	x	x
speechpathology.com [75]	✓	✓	✓	x	x	x	x	X	x	x
medicalnewstoday.com [76]	✓	✓	✓	✓	✓	✓	x	✓ Limited to tubes	x	x
physio-pedia.com [77]	X	✓	x	x	x	x	✓	✓	x	x
SpeechTherapyPD.com [78]	X	✓	X	✓	x	x	✓	x	x	x
EVMS medical group [79]	✓	✓	✓	✓	x	x	x	✓	x	x
Life with a laryngectomy: Vimeo [80]	X	✓	✓	x	✓	✓	x	✓ HME only	✓	x
nnuh.nhs.uk [81]	*	*	*	*	*	*	*	*	*	*
Davis [82]	✓	✓	✓	x	x	x	✓ Emergency only	✓	✓	✓
McDonough [83]	**	**	**	**	**	**	**	**	**	**
Mosters-Benoit [84]	**	**	**	**	**	**	**	**	**	**
Truman [85]	X	X	X	x	x	x	x	✓ Suction only	x	✓
Kashat [86]	**	**	**	**	**	**	**	**	**	**
Sethia [87]	**	**	**	**	**	**	**	✓	✓ Limited to one scenario	✓ Limited to one scenario
Silver [88]	**	✓	✓	**	**	**	**	✓	✓	✓
Torkaz [89]	✓	**	**	**	**	**	**	✓	x	✓ Limited to one scenario
Mughal [90]	**	**	**	**	**	**	**	**	**	**
Wakelam [91]	**	**	**	**	**	**	**	**	**	**
Hsieh [92]	**	**	**	**	**	**	**	**	**	✓ Limited to one scenario

Key:

✓, included

x, not included

*, not specified as depends on training accessed/designed/requested

**, limited information available to determine whether content is included

Twenty-three education/training resources included comprehensive information on laryngectomy anatomy and physiology [46–48, 50, 51, 53–56, 60, 62, 64–68, 70, 74–76, 79, 82, 89]. Thirty-four of the resources contained information on routine airway maintenance and management [46–56, 58–62, 64, 70, 73–80, 83, 88]. However, the level of detail provided varied significantly; some resources provided no information, others provided details about different airway protection products, and others included detailed information, e.g., airway protection products and routine management of the stoma including skin integrity. Similarly, communication and SVR-specific information was detailed in 22 [44, 46–48, 51–53, 57, 60–62, 66, 68, 69, 73–76, 79, 80, 82, 88] and 15 resources [46, 51, 53, 57, 60, 62, 64–67, 69, 74, 77, 78, 82]. respectively, but again, content and detail varied greatly. Twenty-four resources did not contain any information on swallowing [44, 45, 47, 49, 50, 52–61, 64–67, 75, 77, 80, 82, 85]. Only eight resources covered psycho-social considerations for the patient [46, 51, 52, 60, 69, 74, 76, 80] and four resources covered psycho-social input for family [46, 51, 76, 80]. The psycho-social content for patients and family was limited to briefly mentioning this as an expected treatment side-effect, with options for counselling and support groups suggested.

Emergency management in the acute [53, 54, 60–62, 64, 67, 73, 82, 85, 87–89, 92] and community setting [46–50, 54, 60, 61, 67, 80, 82, 87–91] was outlined in 14 resources, with noted variation in the content and detail provided. For example, most resources developed since the NTSP training was rolled out in the UK included reference to the NTSP emergency algorithm [35] and few resources included additional information on emergency scenarios beyond the NTSP information.

### Accessibility

Twenty-eight of the training/education resources were freely accessible online [44–63, 68, 69, 74–77, 79, 80], with 15 only being available only to staff/students at the hosting healthcare facility/University [64, 66, 72, 73, 81–83, 85–92], and three being accessible only by membership [65, 70, 78]. The remaining education/training resources were either in development (*n* = 1) [67] or inaccessible beyond the basic content available (*n* = 2) [74, 84. Twenty-six training resources were free of charge [44–61, 64, 74, 76, 77, 79–81, 85], ten carried a cost to the user [62, 65–70, 72, 75, 78], and whether there was a cost was unknown for 13 resources [63, 71, 73, 82–84, 86, 92]. When a cost was associated with the resource, this ranged from $40 to $1040.

### Certification and Evaluation

Thirty-seven education/training resources were not certified [44–57, 60, 61, 64, 66, 67, 71, 73, 76, 77, 79–92]. Thirty-three training/education resources had not been reviewed since initial publication which ranged from 2012 to 2023 [44–46, 48–50, 52–58, 61–64, 66–73, 75–81, 84]. Only ten resources have been reviewed to date [47, 51, 59, 60, 65, 85, 89–94]; the review of six resources was unclear [74, 82, 83, 86–88].

### Patient and public Involvement (PPI)

Only eight resources had PPI involvement in their education/training [52, 61, 64, 67–69, 73, 80]. For these resources, patient representatives were included in the training delivery, with two resources explicitly also involving patients in content development [67, 73].

## Discussion

This is the first study to use an environmental scan to systematically and comprehensively identify and better understand laryngectomy education and training resources currently available for community HCPs. This study identified 49 resources with applicability to the community, but few specifically target HCPs working predominantly in community settings. All resources included appeared to have a common aim of improving education, training, and awareness of laryngectomy care across multiple parameters. The parameters resources predominantly focussed on included laryngectomy anatomy and physiology, routine airway management, and communication, with other laryngectomy-specific areas such as swallowing, psycho-social support, SVR, and emergency management being less present. There is no uniform approach to the resources, with multiple resources all employing different ways of presenting information, e.g., videos, e-learning modules, written information, and simulation. This results in no consistency, lack of evaluation, and raises questions around the effectiveness of the current education and training, as well as challenges with seeing how the resources would be effectively implemented to improve the safety and care of laryngectomy patients at home.

Laryngectomy care is complex so education and training resources for community HCPs need to be comprehensive and include information on key laryngectomy areas: anatomy and physiology, routine airway maintenance and management, communication, swallowing, psycho-social impact for the person and the family, SVR, equipment, and emergency care applicable to community environments. Only one [50] of the 49 resources in this scan included information on all these areas, but some of the information in this resource is now outdated and thus irrelevant to the current landscape of laryngectomy care, e.g., the use of fax to communicate and use in emergency situations. This links directly to reports in the qualitative literature of poor patient-reported experience of community laryngectomy care [6, 93, 94], with rates as high as 43% of people with laryngectomy feeling that community HCPs do not have the knowledge, or skills required to appropriately manage their needs [22].

Few of the resources identified and reviewed have been specifically designed for community professionals, with most resources either targeting HCPs in general or not stating a target group. It is therefore unlikely that the resources have directly influenced a change in clinicians’ behaviour or clinical intervention to people with a laryngectomy in the community, as very few resources appear relevant to community HCPs as target users. This is supported by the clinician-reported experience literature with frontline clinicians, nurses, and pharmacists all reporting a lack of knowledge, skills, and training to support people with a laryngectomy [19–21, 95]. It is vital that HCPs’ expertise alongside the patient’s voice and experience is included when developing future laryngectomy education and training resources to ensure resources maintain relevance and a practical approach [96]. This could improve community HCP access to laryngectomy-specific training, thus having a positive impact on their knowledge and skills and subsequent patient care and safety in the community.

There was a range of delivery methods described in the education and training resources reviewed, but resources typically relied upon only a single type of training with video, information, or instructor-led being the most popular choice. Whilst theories around learning styles such as auditory, kinaesthetic, and visual have been used frequently in training and education, this theory, referred to as VARK (visual, auditory, reading, kinaesthetic) [97], has been thoroughly debunked in pedagogic literature [98]. This is because there is no adequate evidence base which validates this theory, particularly around the argument that catering to preferred learning styles results in better learning [99]. The delivery methods of most laryngectomy education and training resources included in this review appear to be based on a flawed theory of learning and further support the earlier stated problem with regard to both patient- and clinician-reported experiences [19–21, 95]. Future education and training resources designed for HCPs working with laryngectomy may consider an alternative approach, such as those which offer a meaning-based approach to learning in which the content of the education and training fits the experience, needs, and ability of its user [99, 100]. For example, understanding what has facilitated successful learning in the past, what tools/resources these facilitators have used, and what factors support engagement in learning opportunities.

## Limitations

Although novel in approach, there are limitations to this study. Only education and training resources available in English were included, potentially excluding resources from non-English speaking countries where rates of laryngeal cancer are high [101]. It is highly likely that most of the local education and training programmes developed by healthcare institutions have not been included. Only an overview of content was available for resources requiring payment. Where possible, email contact was made with the lead named author but not all those who were contacted responded.

## Future Directions

Some elements of laryngectomy education, such as those cited in this review, could be offered to community HCPs as standard which may have a valuable impact on improving HCP knowledge and skills, as well as supporting people with a laryngectomy in the community more effectively. New resources could incorporate the lived experience of people with a laryngectomy and their families from the outset through utilizing an experience-based co-design [102] approach which has been successfully used in other studies to improve patient care in cancer [103, 104]. Future research could focus on aiming to scope out the number, content, and impact of organisation-specific laryngectomy education and training to give a better understanding of these resources.

## Conclusions

This environmental scan has provided a detailed snapshot of the laryngectomy education and training environment in 2023–2024. Despite all resources sharing a common aim of improving laryngectomy education, training, and care, the resources included in this scan do not appear to be fully meeting this aim through a lack of consistency in resource content, delivery, evaluation, and implementation. It is evident that the development of community-specific laryngectomy education and training, with clear implementation and evaluation, is required.

## Data Availability

The dataset analyzed during the current study is available at request of the lead author.
